# Global Metabolic Profiling of Baculovirus Infection in Silkworm Hemolymph Shows the Importance of Amino-Acid Metabolism

**DOI:** 10.3390/v13050841

**Published:** 2021-05-06

**Authors:** Min Feng, Shigang Fei, Junming Xia, Mengmeng Zhang, Hongyun Wu, Luc Swevers, Jingchen Sun

**Affiliations:** 1 Guangdong Provincial Key Laboratory of Agro-animal Genomics and Molecular Breeding, College of Animal Science, South China Agricultural University, Guangzhou 510642, China; hunanfengmin@163.com (M.F.); fsg514696098@163.com (S.F.); xiajm2018@163.com (J.X.); Gcy1053462127@163.com (M.Z.); hyhyhyhyw@163.com (H.W.); 2 Insect Molecular Genetics and Biotechnology, National Centre for Scientific Research Demokritos, Institute of Biosciences and Applications, 15310 Athens, Greece

**Keywords:** baculoviruses, *Bombyx mori*, metabolome, BmNPV, amino acid

## Abstract

Viruses rely on host cell metabolism to provide the necessary energy and biosynthetic precursors for successful viral replication. Infection of the silkworm, *Bombyx mori*, by *Bombyx mori* nucleopolyhedrovirus (BmNPV), has been studied extensively in the past to unravel interactions between baculoviruses and their lepidopteran hosts. To understand the interaction between the host metabolic responses and BmNPV infection, we analyzed global metabolic changes associated with BmNPV infection in silkworm hemolymph. Our metabolic profiling data suggests that amino acid metabolism is strikingly altered during a time course of BmNPV infection. Amino acid consumption is increased during BmNPV infection at 24 h post infection (hpi), but their abundance recovered at 72 hpi. Central carbon metabolism, on the other hand, particularly glycolysis and glutaminolysis, did not show obvious changes during BmNPV infection. Pharmacologically inhibiting the glycolytic pathway and glutaminolysis also failed to reduce BmNPV replication, revealing that glycolysis and glutaminolysis are not essential during BmNPV infection. This study reveals a unique amino acid utilization process that is implemented during BmNPV infection. Our metabolomic analysis of BmNPV-infected silkworm provides insights as to how baculoviruses induce alterations in host metabolism during systemic infection.

## 1. Introduction

Viruses are considered the ultimate parasites, and the materials needed for their life activities must be provided by their host. Viruses typically depend on the host cell to obtain the building blocks and biosynthesis machinery needed for virion replication and assembly [1,2,3]. A large amount of new knowledge has been obtained on the interaction between virus and host through mRNA transcriptome and proteome analysis, but these approaches do not directly describe the use of cellular metabolites by viruses. There is no doubt that viral infection triggers metabolic reprogramming in host cells to facilitate virus production [1,2,3]. As a new research field, the mechanisms and consequences of virus-induced metabolic reprogramming have attracted more and more researchers’ attention.

Fortunately, high-throughput technologies for the analysis of metabolic alterations in host cells (“metabolomics”) have recently become widely available and have greatly expanded our knowledge of these crucial host-viruses interactions [3,4]. For instance, Zika virus infection resulted in enhanced glucose utilization through the tricarboxylic acid (TCA) cycle in human cells, whereas this process was shifted toward the pentose phosphate pathway in mosquito cells [5]. Newcastle disease virus infection increased pools of amino acids and nucleotides to benefit viral protein synthesis and genome amplification [6]. Influenza A virus regulates the abundance of host substances related to purine, lipid, and glutathione metabolism during early infection to provide the energy and components for efficient completion of its replication cycle [7]. Indeed, both DNA and RNA viruses have been shown to reprogram various aspects of metabolism, including increased glycolysis, elevated pentose phosphate activity to support amino acid generation, production of nucleotides, and lipid synthesis [2,3]. However, while there are common metabolic changes induced by most viruses, often unique characteristics also exist necessitating the study of each virus species individually. Specifically, the role of metabolic reprogramming in the pathogenesis of viral diseases is still not clear in many instances.

Baculoviruses are invertebrate-specific circular double-stranded DNA viruses with genome sizes varying from about 80 to over 180 kb, which bring harm to economically important (mainly lepidopteran) insects but also are applied as bio-insecticides in pest control and used as biotechnological platforms for the expression of heterologous proteins [8]. *Bombyx mori* nucleopolyhedrovirus (BmNPV) is a representative member of baculoviruses that specifically infects silkworms and causes serious losses in sericulture industry [8,9]. Like other baculoviruses, BmNPV produces two types of virions during the infection cycle, i.e., the occlusion-derived virus (ODV) and the budded virus (BV) [8]. ODVs are occluded within a crystalline protein matrix, known as occlusion bodies, which transmit viruses from silkworm to silkworm via oral infection, whereas BVs spread viruses from cell to cell within animals [8]. The genome of BmNPV is very large with a size of 130 kb and contains 136 putative open reading frames [10]. Therefore, BmNPV replication inevitably necessitates the availability of a large pool of host metabolites such as nucleotides and amino-acids as well as adequate sources of energy. However, how baculoviruses reprogram host metabolism during natural infections of silkworms remains unclear.

As the model species of Lepidoptera and the only truly domesticated insect, the domesticated silkworm has been used for basic and applied research for a long time. To establish how metabolism and its underlying transcriptional regulation are rewired during DNA virus/baculovirus infection, we have constructed a global metabolite-gene network based on a metabolome and transcriptome dataset of hemolymph samples taken at different stages of BmNPV infection. Co-expression analysis that integrates metabolome and transcriptome data provided interesting correlations between major metabolites and gene expression during BmNPV infection. Using this dataset, key regulatory networks for major metabolites were discovered in which new key genes were associated with important metabolites. Our results therefore will provide a new perspective on baculovirus-host interaction and can be considered as a useful resource for identifying key regulators of important metabolic pathways for other baculovirus-insect infection models.

## 2. Materials and Methods

### 2.1. Silkworm and Virus Infection

Larvae of silkworm (*B. mori*, Dazao P50 strain) were reared with fresh mulberry (*Morus* sp.) leaves and reared at a temperature of 28 °C and humidity between 60 and 70%. Recombinant BmNPV-EGFP, as a reporter virus, was constructed by BmNPV-based Bac-to-Bac System (*Bombyx mori* MultiBac) [11] of the Guangdong Provincial Key Laboratory of Agro-animal Genomics and Molecular Breeding. Newly molted fifth-instar silkworm larvae were injected with either 10 μL of BmNPV-EGFP (10^4.8^ TCID_50_) or PBS (Negative Control, NC). Blue Light Gel Imager (Sangon Biotech, China) was used to monitor the spread of green fluorescence in infected larvae on a daily basis. Viral load and mortality were monitored from 1 day post-infection (dpi) to 5 dpi within 5 groups (25 animals/group). Viral load in hemocytes was detected by absolute quantitation [12] using qPCR with *gp41* primer pair [13].

### 2.2. Sample Preparation

Hemolymph samples were collected from silkworms by bleeding at 1 day and 3 days after BmNPV or PBS treatment. The hemolymph mixtures were centrifuged at 4000× *g* for 10 min at 4 °C to separate supernatant and hemocytes. The supernatant and hemocytes were used for Metabolomic and RNA sequencing (RNA-seq) analysis, respectively.

For metabolomic samples, the supernatants of hemolymph collected from six silkworms were mixed as one repeat sample. Each of the four experimental groups contained 6 replicates (6 larvae/replicate) and were named BmNPV-1d, NC-1d, BmNPV-3d, and NC-3d. Correspondingly, the hemocyte pellets collected from six replicates (36 larvae) of the BmNPV-1d, NC-1d, BmNPV-3d, and NC-3d groups were used as RNA-seq samples. The supernatant and hemocyte pellet samples were frozen by immersion in liquid nitrogen and stored at 80 °C until metabolomic or RNA-seq analysis. Metabolomic and RNA-seq analysis were performed by Gene denovo Biotechnology Co., Ltd. (Guangzhou, China).

### 2.3. Metabolite Extraction

The amount of 100 μL of each supernatant of hemolymph sample was transferred to an eppendorf tube. After the addition of 400 μL of extract solution (acetonitrile: methanol = 1:1, containing isotopically-labelled internal standard mixture), the samples were vortexed for 30 s, sonicated for 5 min in an ice-water bath, and incubated for 1 h at −20 °C to precipitate proteins. Then, the samples were centrifuged at 12,000× *g* rpm for 15 min at 4 °C (Heraeus Fresco 17, Thermo, USA). The resulting supernatant was transferred to a fresh glass vial for analysis. The quality control (QC) sample was prepared by mixing an equal aliquot of the supernatants from all samples.

### 2.4. Liquid Chromatography-Tandem Mass Spectrometry (LC-MS/MS) Analysis

LC-MS/MS analyses were performed using an UHPLC system (Infinity 1290, Agilent Tech., CA, USA) with a UPLC HSS T3 column (1.7 μm, 2.1 × 100 mm, Waters) coupled to a Q Extractive™ Orbitrap Mass Spectrometer (Thermo, USA) in electrospray ionization (ESI) positive (POS) and negative (NEG) ion mode. The mobile phase consisted of a mixture of 25 mM ammonium acetate and 25 mM ammonia hydroxide in water (pH = 9.75) and acetonitrile (ACN). The analysis was carried out with an elution gradient as follows: 0~0.5 min, 95% ACN; 0.5~7.0 min, 95%~65% ACN; 7.0~8.0 min, 65%~40% ACN; 8.0~9.0 min, 40% ACN; 9.0~9.1 min, 40%~95% ACN; 9.1~12.0 min, 95% ACN. The column temperature was 25 °C. The auto-sampler temperature was 4 °C and the injection volume was 3 μL.

The information-dependent acquisition (IDA) mode of the mass spectrometer was used to acquire MS/MS spectra of 1 μL injection aliquots. In IDA mode, the acquisition software Xcalibur4.0.27 (Thermo, USA) was applied continuously to survey and evaluate the full scan of MS data, as it acquires MS/MS spectra depending on preselected criteria. ESI conditions were set as follows: sheath gas flow rate = 45 arb, auxiliary gas flow rate = 15 arb, capillary temperature = 400 °C, full MS resolution = 70,000, MS/MS resolution = 17,500, collision energy = 10/30/60 eV in NCE (Normalized Collision Energy) model, spray voltage = 4.0 kV (positive mode) or −3.6 kV (negative mode), respectively.

### 2.5. Processing and Statistical Analysis of Metabolomics Data

The R package XCMS (version 3.2) was used to analyze the raw data for peak alignment, calibration, and retention time peak area extraction. In addition, the internal standard normalization method was used in the data analysis. A data matrix was generated consisting of the sample information, peak retention time (RT), mass to charge ratio (M/Z), and peak intensity. OSI-SMMS (version 1.0, Dalian Chem Data Solution Information Technology Co. Ltd.), incorporated with MassBank, HMDB, METLIN, mzcloud database, and an in-house MS/MS database, was used for peak annotation. Multidimensional statistical analysis including principle component analysis (PCA), partial least squares-discriminant analysis (PLS-DA), and orthogonal partial least squares discriminant analysis (OPLS-DA) were further used to show the distribution of original data and classification of variables.

The characteristics of metabolite expression patterns were used to identify the differential abundance of metabolites between BmNPV-infected and control samples using the Variable Importance for the Projection (VIP) analysis of OPLS-DA data with the criteria VIP ≥ 1 and *p* < 0.05 in Student’s t test thresholds. The differential metabolites were further analyzed for pathway enrichment according to the Kyoto Encyclopedia of Genes and Genomes (KEGG) Metabolome Database.

### 2.6. Generation of Transcriptome Profiling

The hemocyte pellets collected from the BmNPV-1d, NC-1d, BmNPV-3d, and NC-3d groups were used to construct mRNA libraries. After total RNA was extracted, mRNA was enriched by binding to Oligo(dT) beads, fragmented into short fragments using fragmentation buffer and reverse transcribed into cDNA with random primers. Second-strand cDNA was synthesized by DNA polymerase I, RNase H, dNTP, and buffer. cDNA fragments were purified with QiaQuick PCR extraction kit (Qiagen, Venlo, The Netherlands), end repaired, poly(A) added and ligated to Illumina sequencing adapters. The ligation products were size selected by agarose gel electrophoresis, PCR amplified, and sequenced using Illumina HiSeq2500 by Gene Denovo Biotechnology Co. (Guangzhou, China).

Transcriptome profiling was also performed by Gene Denovo Biotechnology Co. (Guangzhou, China). Briefly, the high-quality clean reads were mapped to the latest version of the silkworm genome (SilkDB3.0) using HISAT2. 2.4 [14]. The mapped reads were assembled using StringTie v1.3.1 [15]. FPKM (fragment per kilobase of transcript per million mapped reads) values were calculated using StringTie software [15] to quantify gene expression abundance and variations. The edgeR package [16] was used to identify differentially expressed genes (DEGs) across groups (BmNPV vs. NC). Genes with a fold change (FC) of |log2FC| >1 and a false discovery rate (FDR) < 0.05 were considered DEGs.

### 2.7. Integrative Analysis of Metabolome and Transcriptome

To identify metabolite-related pathways associated with baculovirus infection that are based on both the metabolome and transcriptome dataset, DEGs and DE metabolites were examined for overlapping metabolic pathways. In addition, Pearson correlation coefficients were calculated for metabolome and transcriptome data integration. Gene and metabolite pairs were ranked in the descending order of the absolute value (positive and negative) of correlation coefficients. The pairs of genes and metabolites in common metabolic pathways (with absolute Pearson correlation coefficient > 0.995 and *p* < 0.05) were visualized using Cytoscape (V3.3.0).

### 2.8. Treatment of BmN Cells with Inhibitors of Glycolysis and Glutamine Metabolism on BmN Cells

Glycolysis inhibitor 2-deoxyglucose (2-DG) [17] and glutamine metabolism inhibitor CB-839 [18] have been found to affect virus replication. To determine an appropriate concentration of 2-DG and CB-839, BmN cells (2.5 × 10^4^ cells/well) were pre-incubated with 2-DG (2, 4, 6, 8, 10, 20, 30, 40 mM, MCE, USA), or CB839 (2, 4, 6, 8, 10 nM, MCE, USA) for 48 h in 48 well-plates. Cytotoxicity was determined using Cell Counting Kit-8 dye (Beyotime, China). In virus infection experiments, BmN cells (1.25 × 10^5^ cells) were pretreated with 2-DG and CB-839 at the optimum concentration for 2 h. Cells pretreated with the same dose of solvent (water and DMSO for 2-DG and CB-839, respectively) were used as control. BmN cells were subsequently infected with BmNPV at 5 MOI for 1 h at 27 °C. Then, the supernatant was replaced with fresh Grace’s Insect Cell Culture Medium (10% FBS) (Thermo, USA) supplemented with 2-DG and CB839 (optimum concentration), respectively. This time point was defined as 0 hpi. Total DNA (cellular DNA and viral DNA) was harvested to detect viral load at 24 and 48 h post-infection (hpi). Viral DNA was detected by absolute quantitation [12] using qPCR with *gp41* primer pair [13]. Experiments were carried out independently three times. Statistical comparisons were performed using GraphPad Prism 8 (GraphPad Software Inc., USA). Results are presented as means ± SEM, and statistical significance was assessed at *p* values of <0.05, 0.01, or 0.001.

## 3. Results

### 3.1. Replication of BmNPV in Silkworm Hemocytes

**Figure 1:**
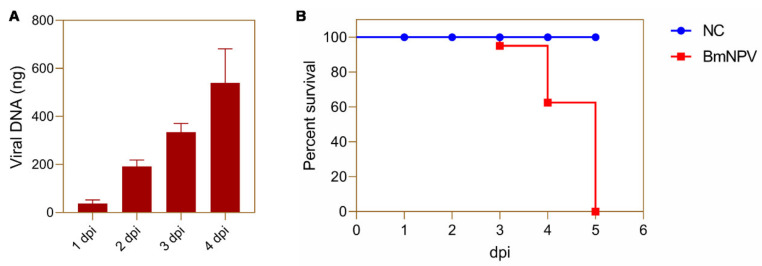
Time course of BmNPV replication and silkworm mortality. Newly molted fifth-instar silkworm larvae (Dazhao) were injected with either 10 μL of BmNPV-EGFP (10^4.8^ TCID50) or PBS. (**A**) Hemocytes were collected for absolute quantification of viral DNA at 1, 2, 3, and 4 d. (**B**) The survival curve of silkworm larvae after injection of BmNPV or PBS for 1–5 days.

To find suitable sampling time points, the concentration of BmNPV DNA was measured in hemocytes. From 1 to 4 dpi, amounts of viral DNA in hemocytes gradually increased (Figure 1A). However, a large number of infected silkworms started to die from the 4th day (Figure 1B). Therefore, 1 and 3 dpi are considered to represent appropriate time points for the early and late stages of BmNPV infection in the present study.

### 3.2. Multivariate Analysis of Silkworm Hemolymph Metabolites

**Figure 2:**
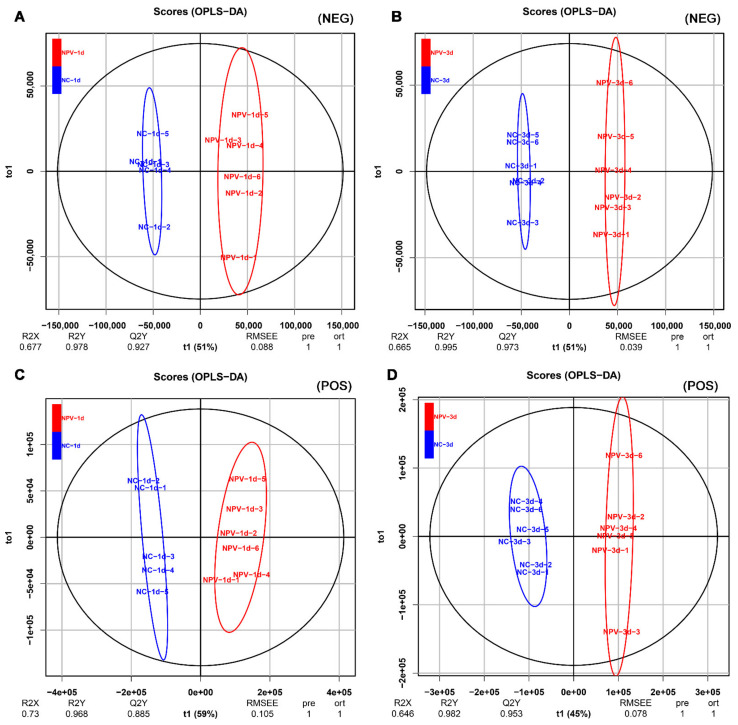
OPLS-DA scores of polar components of hemolymph metabolic profiling analysis. The OPLS-DA models (A, B, C, D) were derived from the LC-MS/MS metabolomic profiles of the hemolymph samples. Both negative mode (NEG) and positive (POS) models are shown for 1 dpi (**A**,**C**) and 3 dpi (**B**,**D**) as indicated.

Both positive and negative ion modes of ESI were used during LC-MS/MS analysis. Data matrices were generated following retention time alignment, peak detection, peak matching, and normalization of the raw data. Based on the OSI-SMMS and KEGG COMPOUND Metabolomics Library, valid peaks were matched for 485 (POS) and 210 (NEG) silkworm hemolymph metabolites. The PCA-score plots displayed tightly clustered QC groups in BmNPV-infected and control samples under both POS and NEG conditions (Figure S1), indicating good analytical reproducibility and reliability of the LC-MS/MS analysis. However, sample NC-1d-6 showed as an obvious outlier with the other five NC-1d samples in POS mode (Figure S1). Therefore, the NC-1d-6 sample in POS mode was removed in the subsequent analysis. Furthermore, multivariate statistical analysis of OPLS-DA models (Figure 2) revealed a clear separation between BmNPV-infected samples and control samples. The R2X, R2Y, and Q2 predictive parameters of OPLS-DA are shown at the bottom of the OPLS-DA image (Figure 2). These results indicate that the silkworm model of BmNPV infection was reliable and reproducible.

### 3.3. Characteristic Metabolites in Response to BmNPV Infection

**Figure 3:**
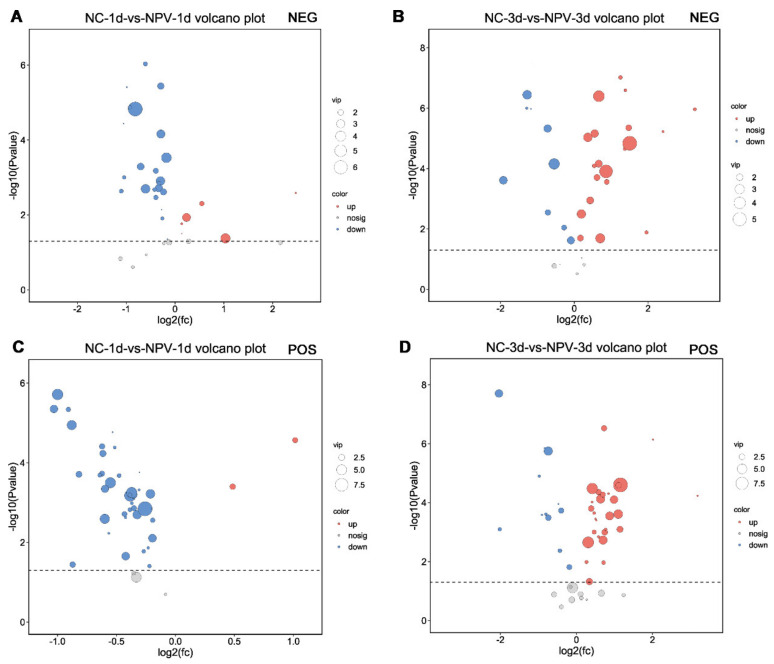
Volcano plots of metabolites in the hemolymph of BmNPV-infected and PBS-injected silkworms. (**A**,**B**) were derived from the negative mode (NEG) models at 1 d and 3 d, respectively. (**C**,**D**) were derived from the positive mode (POS) models at 1 d and 3 d, respectively. Each point in the volcanic map represents a metabolite. Red: Up-regulated metabolites. Blue: Down-regulated metabolites. Gray: Not significant. fc: Fold change.

OPLS-DA and univariate analysis were performed to analyze the total metabolite profiles in BmNPV-infected and control samples. The VIP analysis in the OPLS-DA model (VIP > 1) and *p*-value of Student’s *t*-test (*p* < 0.05) were used as criteria to screen metabolites with differential abundance. Volcano plots in Figure 3 show all the differentially expressed (DE) metabolites, which were identified in silkworm hemolymph at one day and three days post-BmNPV infection.

**Figure 4:**
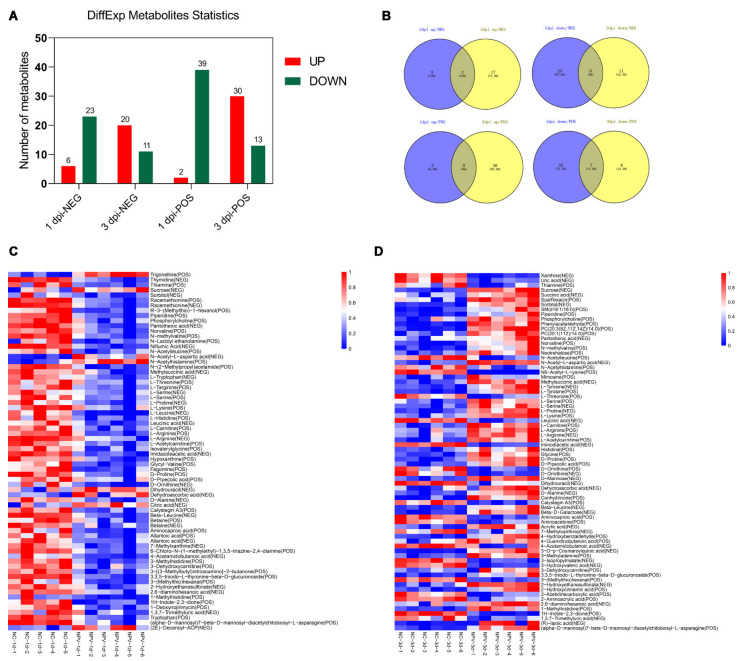
Analysis of differentially expressed (DE) metabolites in hemolymph of BmNPV-infected and uninfected silkworms. (**A**) Numbers of metabolites that were up-regulated (red) and down-regulated (green) in BmNPV-infected silkworm larvae. (**B**) Venn diagrams provide a global overview of the common and DE of metabolites between the 1 d and 3 d groups. (**C**,**D**) Heatmaps of DE metabolites in hemolymph at 1 d (**C**) and 3 d (**D**) after BmNPV infection. Each column represents one sample, and each row represents one DE metabolite. Red color represents the relative level of the up-regulated metabolites, and blue color represents down-regulation.

Using statistics based on metabolite identity determined by analysis of both NEG and POS mode, a total of 144 metabolites in hemolymph were significantly changed after BmNPV infection, corresponding to 70 and 74 significantly changed metabolites at 1 dpi and 3 dpi, respectively (Supplementary Data Sheet S1). A total of 8 and 50 metabolites were significantly up-regulated at 1 and 3 dpi while 62 and 24 metabolites were significantly down-regulated at these time points (Figure 4A). An overview of the distribution of common and DE metabolites at 1 and 3 days is presented as Venn diagrams (Figure 4B). Only three metabolites (N-acetyl-L-aspartic acid, dehydroascorbic acid, and sucrose) were significantly up-regulated at both time points, while seven metabolites (thiamine, L-threonine, N-acetylleucine, 3-(methylthio)hexanal, aminocaproic acid, calystegin A3, and 1H-indole-2,3-dione) were significantly down-regulated at both 1 and 3 dpi (Figure 4B). To further observe patterns of overall metabolite abundance, heat map analysis was performed which clearly displayed a significant difference in the abundance of metabolites between 1 and 3 dpi (Figure 4C,D). After 1 day of infection, most metabolites were significantly down-regulated compared to the control samples (Figure 4 A, C). On the other hand, the majority of metabolites was significantly up-regulated at 3 dpi compared to uninfected silkworms (Figure 4A,D).

### 3.4. Amino Acid Metabolism Pathways Are Significantly Activated during BmNPV Infection

**Figure 5:**
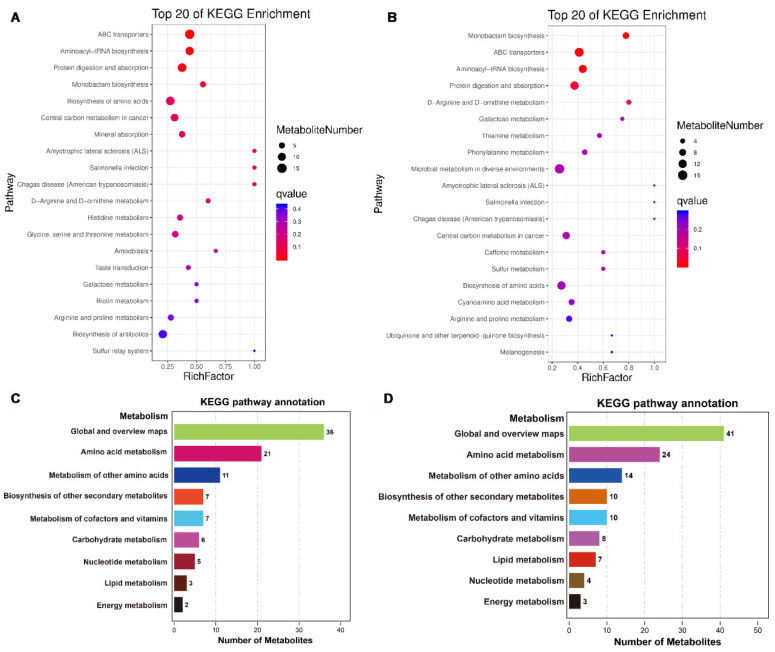
Diagram of metabolic pathways in hemolymph affected by BmNPV infection. (**A**,**B**) Top 20 KEGG-enriched pathways based on DE metabolites in the hemolymph of BmNPV-infected silkworm at 1 (**A**) and 3 dpi (**B**). The statistics of metabolites enriched in the KEGG class metabolism at 1 dpi (**C**) and 3 dpi (**D**).

To determine important functional networks upon BmNPV infection in silkworm hemolymph, metabolic pathway analysis was performed with respect to the significantly changed metabolites at 1 and 3 dpi (Supplementary Data Sheet S2). The top 20 pathways activated by BmNPV infection are presented in Figure 5A,B. During the early stage of BmNPV infection (1 dpi), amino acid metabolism-related pathways such as ABC transporters, aminoacyl-tRNA biosynthesis, protein digestion and absorption, biosynthesis of amino acids, D-arginine and D-ornithine metabolism, histidine metabolism and glycine, serine, and threonine metabolism were highly associated with the response to BmNPV infection (Figure 5A). On the other hand, amino acid metabolism-related pathways such as ABC transporters, aminoacyl-tRNA biosynthesis, protein digestion and absorption, D-arginine, and D-ornithine metabolism, and biosynthesis of amino acids, were also highly associated with the response to BmNPV infection at 3 dpi (Figure 5B). With regard to all DE metabolites, the number of significantly differentially expressed amino acid metabolites is prevalent after BmNPV infection at 1 and 3 dpi (Figure 5 C, D). In summary, the amino acid metabolism pathway seems to be the most severely affected by BmNPV infection at both early and late time points.

In mammalian cells, both virus-infected cells and cancer cells commonly exhibit the Warburg effect, i.e., an increase in both glucose catabolism and lactate production independent of oxygen concentration [2]. The Warburg effect was also observed during tumor growth in *Drosophila* and is detected during activation of the immune response in insect hemocytes [19,20]. Therefore, the KEGG category “central carbon metabolism in cancer pathway” that was enriched during BmNPV infection at 1 and 3 dpi also caught our attention since it could suggest the occurrence of similar metabolic reprogramming (Figure 5A, B). However, the metabolites that were enriched in the central carbon metabolism pathway in the hemolymph of BmNPV-infected cells were almost all amino acids with only citric acid and succinic acid (both are components of the TCA cycle) as exceptions. In addition, increased lactate production, indicative of activation of hemocytes in *Drosophila* [2], was not observed in our analysis.

### 3.5. Accumulation and Depletion of Individual Key Metabolites at Different Times by BmNPV Infection

**Figure 6:**
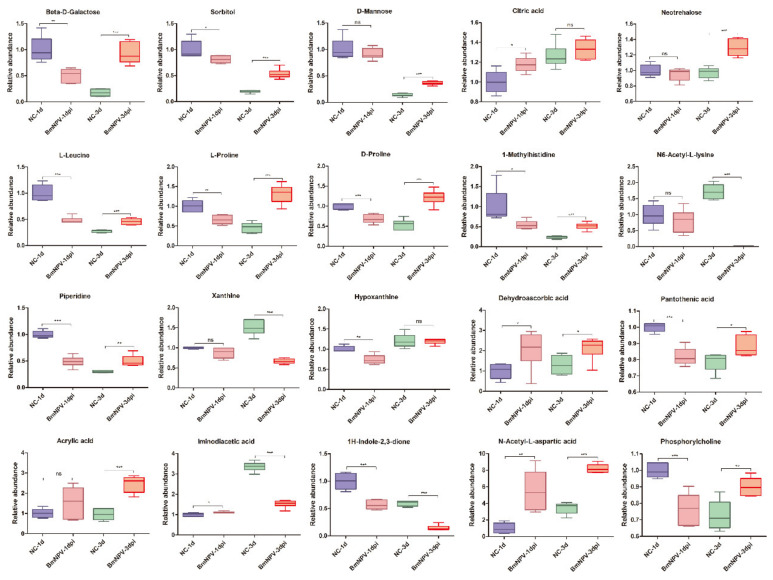
Abundance analysis of key metabolites that showed significant changes during BmNPV infection. The data shown are mean ± SE (*n* = 5 or 6). Statistical comparisons were performed using GraphPad Prism 8 (GraphPad Software Inc., USA). Statistical significance was assessed at *p* values of <0.05, 0.01, or 0.001. ns *p* > 0.05.

A more detailed analysis of changes in the levels of DE amino acids, carbohydrates, and nucleotides in silkworm hemolymph at 1 and 3 dpi is presented in Figures S2–S4, respectively. On the whole, the abundance of DE amino acids was reduced after BmNPV infection at 1 dpi and increased at 3 dpi (Figure S2). DE carbohydrates were significantly up-regulated after BmNPV infection at 3 dpi (Figure S3). Strikingly, the abundance of most metabolites that show significant changes such as beta-D-galactose, sorbitol, D-mannose, L-leucine, proline, piperidine, glycine, arginine, serine, pantothenic acid, and phosphorylcholine, exhibit a similar pattern, i.e., a significant reduction at 1 dpi followed by a notable increase at 3 dpi (Figure 6). A divergence in this pattern was observed for citric acid, dehydroascorbic acid, and N-acetyl-L-aspartic-acid that displayed an upregulation of their abundance at 1 dpi (Figure 6). In addition, the abundance of N6-acetyl-L-lysine, xanthine, iminodiacetic-acid, and 1H-indole-2,3-dione was down-regulated at 3 dpi (Figure 6).

### 3.6. Analysis of Differentially Expressed Genes in Silkworm Hemocytes after BmNPV Infection

**Figure 7:**
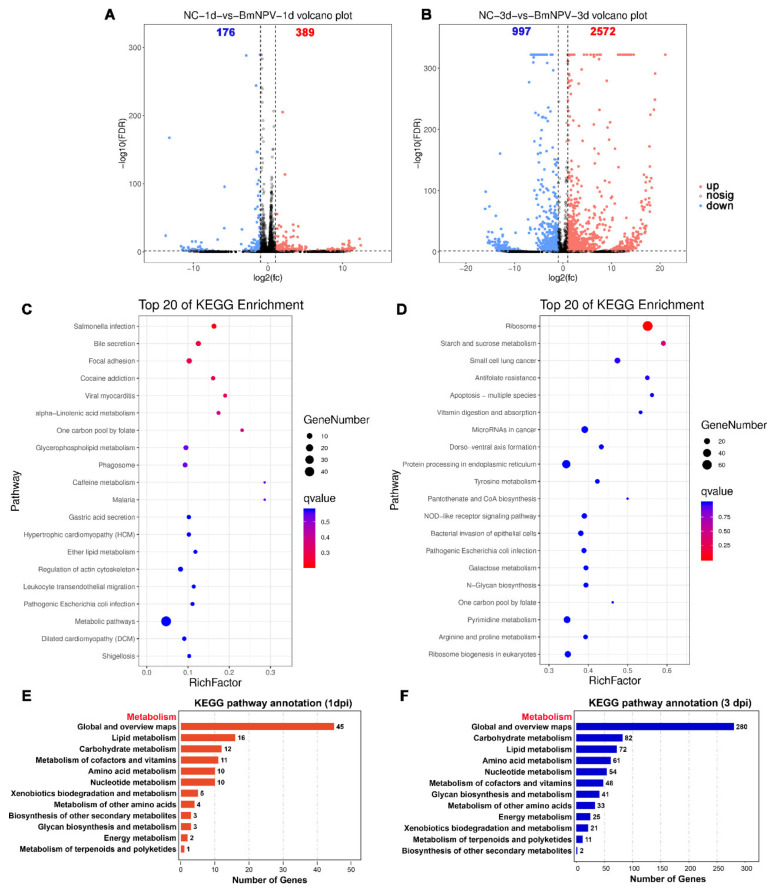
Analysis of DEGs in silkworm hemocytes after BmNPV infection. (**A**,**B**) Volcano plots of changes in gene expression in BmNPV-infected samples in comparison with uninfected hemocytes at 1 (**A**) and 3 dpi (**B**), respectively. Each point in the volcanic map represents a gene. Red: Up-regulated genes. Blue: Down-regulated genes. Gray: Not significant. (**C**,**D**) Top 20 KEGG-enriched pathways of DEGs in hemocytes of BmNPV-infected silkworm larvae at 1 (**C**) and 3 dpi (**D**). (**E**,**F**) The statistics of genes enriched in the KEGG class metabolism at 1 dpi (**E**) and 3 dpi (**F**).

To investigate the corresponding transcriptional regulation to the metabolite changes during BmNPV infection, we built a transcriptome landscape of silkworm hemocytes at 1 and 3 d post-virus infection. We found that at 1 and 3 dpi, 389 and 2572 DEGs were up-regulated, and 176 and 997 DEGs were down-regulated in BmNPV-infected hemocytes (Figure 7A,B). Among these DEGs, 170 genes were induced at both 1 and 3 dpi (Figure S5A). In addition, the expression of 56 genes was inhibited in hemocytes after BmNPV infection at both 1 and 3 dpi (Figure S5B). KEGG analysis illustrates that DEGs induced by BmNPV at 1 dpi were significantly enriched in metabolism-related pathways that include alpha-linolenic acid metabolism, one carbon pool by folate, glycerophospholipid metabolism, caffeine metabolism, ether lipid metabolism, and metabolic pathways (*p* < 0.05) (Figure 7C). DEGs identified at 3 dpi were significantly enriched in the categories starch and sucrose metabolism and vitamin digestion and absorption (*p* < 0.05) (Figure 7D). In contrast to the observation that DE metabolites were mainly enriched in amino acid metabolism pathways (metabolome analysis), DEGs at 1 dpi were mainly enriched in lipid metabolism and carbohydrate metabolism (transcriptome analysis) (Figure 7E), whereas DEGs at 3 dpi were mainly enriched in carbohydrate metabolism, lipid metabolism, amino acid metabolism, and nucleotide metabolism (Figure 7F). Actually, after three days of baculovirus infection, a large number of DEGs involving a wide range of metabolic pathways was affected (Figure 7F).

However, the metabolic pathways enriched by DE metabolites are very different from the metabolic pathways enriched by DEGs. In the top 20 of KEGG enrichment pathways, only caffeine metabolism and porphyin and chlorophyll metabolism are simultaneously enriched in both DE metabolites and DEGs at 1 dpi (Figure S6A). At three days post-BmNPV infection, galactose metabolism, arginine, and proline metabolism and glutathione metabolism are the metabolic pathways that are enriched in common by DE metabolites and DEGs (Figure S6B).

### 3.7. Integrated Analysis of Metabolome and Transcriptome

To understand the regulatory network of genes and metabolites during BmNPV infection, we carried out Pearson correlation tests between quantitative changes of metabolites and transcripts. The gene–metabolite correlation pairs with absolute Pearson correlation coefficient > 0.995 and *p* < 0.05 were used to build regulatory networks.

The resulting network of amino acid metabolism consisted of 757 transcripts and 56 metabolites (Figure S7, Supplementary Data Sheet S3). Metabolites in the category of amino acid metabolism that include 3-isopropylmalate, 2-hydroxycinnamic acid, N6-acetyl-L-lysine, L-threonine, aminoacetone, histamine, and 4-guanidinobutanoic acid were significantly correlated with a large number of different transcripts (Figure S7). Regarding carbohydrate metabolism, the analysis revealed a strong correlation of 366 transcripts with 41 metabolites (Figure S8, Supplementary Data Sheet S3). Sucrose showed the highest degree of correlation with 237 differentially expressed transcripts (Figure S8). In the category nucleotide metabolism network, 117 transcripts had strong correlations with 39 metabolites (Figure S9, Supplementary Data Sheet S3). Uric acid, thymidine, and xanthine were found to be most densely connected to a large number of DEGs (Figure S9). In addition, many DEGs (indicated with red color) showed strong correlations within the networks of purine metabolism and pyrimidine metabolism (Figure S9). The regulatory network of lipid metabolism consisted of 340 transcripts and 35 metabolites (Figure S10, Supplementary Data Sheet S3) in which the glycerophospholipids PC(20:3(8Z,11Z,14Z)/14:0) and PC(20:1(11Z)/14:0) showed the highest density of connections with DEGs (Figure S10).

**Figure 8:**
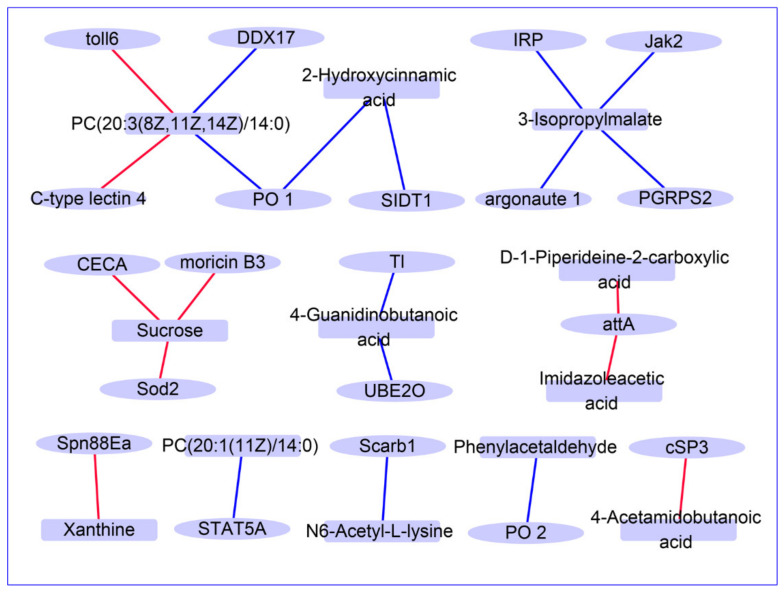
The network diagram of immune-related DE metabolite-DEG pairs. The ovals represent the differentially expressed (DE) metabolites, and the boxes represent the differentially expressed genes (DEGs). The red line connects the positive correlation pair, and the blue line connects the negatively correlated DE metabolite-DEG pairs.

Furthermore, 22 immune related gene-metabolite pairs were screened, which is presented in Figure 8. Twenty immune related genes such as *attA*, *CECA*, *Jak2*, *moricin B3*, *PGRPS2*, *argonaute 1*, and twelve metabolites such as 3-isopropylmalate, sucrose, and xanthine, could be associated as DEG-DE metabolite pairs (Figure 8). These results indicate that changes in metabolites may also be related to the antiviral immune response during BmNPV infection.

### 3.8. Glycolysis and Glutaminolysis May Not Play a Key Role in the BmNPV Infection Process

Glucose and glutamine are utilized as essential sources of carbon for production of energy and synthesis of macromolecules. Glycolysis and glutaminolysis have been shown to be necessary for the replication of multiple viruses [1,17,21,22]. However, in the present study, several key metabolites involved in glycolysis, TCA cycle, and glutaminolysis, such as glutamine, glutamic acid, aspartic acid, glucose, α-ketoglutarate, acetyl-CoA, and oxaloacetic acid, were not detected or their abundance was not affected by BmNPV infection (Supplementary Data Sheet S4). Only citric acid (at 1 dpi) and succinic acid (at 3 dpi) in the TCA cycle were significantly increased after BmNPV infection (Supplementary Data Sheet S4) and changes in abundance were small. In addition, both glutamine transporter- and glucose transporter-related genes were expressed at low levels and were not induced by BmNPV infection in hemocytes (Supplementary Data Sheet S4). These results suggest that glutamine catabolism, glycolysis, and TCA cycle were not activated by BmNPV infection.

### 3.9. Glycolysis and Glutaminolysis Inhibitors Do Not Inhibit BmNPV Replication

**Figure 9:**
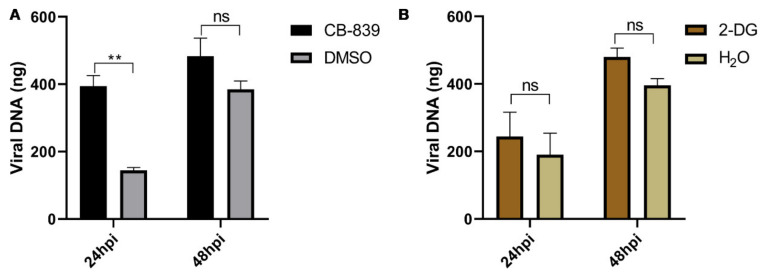
Inhibition of glycolysis and glutaminolysis does not inhibit BmNPV replication. BmN cells (1.25 × 10^5^) were pretreated with CB-839 (8 nM) (**A**) or 2-DG (6 mM) (**B**) for 2 h. Cells pretreated with ddH2O or DMSO (0.0001%) were used as control. BmN cells were subsequently infected with BmNPV at 5 MOI for 1 h at 27 °C. Then, the supernatant was replaced with fresh Grace’s Insect Cell Culture Medium (10% FBS) supplemented with 2-DG and CB-839, respectively (0 hpi). Total DNA was harvested to detect viral load at 24 and 48 hpi. This experiment was performed independently three times. Statistical comparisons were performed using GraphPad Prism 8 (GraphPad Software Inc., USA). Results are presented as means ± SEM. ns *p* > 0.05, ** *p* < 0.01.

To determine the impact of glycolysis and glutaminolysis on BmNPV infection, we treated BmNPV-infected BmN cells with the glycolytic inhibitor 2-DG (6 mM) and glutaminolysis inhibitor CB-839 (8 nM) at their optimum concentrations (Figure S11). As shown in Figure 9, treatment of BmN cells with the glutaminolysis inhibitor CB-839 dramatically increased BmNPV replication at 24 hpi (Figure 9A). However, treatment of BmN cells with glycolytic inhibitor 2-DG had no significant effect on BmNPV replication at 24 and 48 hpi (Figure 9B). Our results showed that inhibition of glutaminolysis enhanced BmNPV replication in vitro, which is contrary to what was observed in other virus infection models [18]. These results indicated that glutaminolysis and glycolysis may not be essential for optimal replication of BmNPV.

## 4. Discussion

In recent years, great progress has been made in the understanding of the important role that metabolic pathways play in the interaction between virus infections and host responses [1,2]. The concept of metabolism as an important parameter in the interaction between host and virus was already raised a considerable time ago but accelerated after the development of high-throughput approaches such as LC-MS [3]. During our study, we focused on the systemic infection of silkworm larvae with BmNPV and identified 65 and 70 metabolites with differential abundance at early and later stages of infection. It can be assumed that changes in the DE metabolites reflect the mechanism by which BmNPV regulates host cell metabolism to benefit its replication. Although the number of DE metabolites is similar at 1 and 3 dpi of BmNPV infection, the trend of their accumulation is strikingly different. Overall, most metabolites were significantly down-regulated after 1 day of infection, whereas an opposite pattern of up-regulation was detected at 3 dpi (Figure 4). BmNPV replication occurs at relatively low levels at 1 dpi (Figure 1) and the virus may start replication by consumption of already-available metabolites in the host cell, therefore causing a decline in metabolite levels, particularly those of amino acids (Figure 4C). As virus replication intensifies at 3 dpi (Figure 1), the virus needs to reprogram cell metabolism to meet the large amount of basic components needed for sustained replication at high levels (Figure 4D).

It is important to realize that the experimental system of this study, BmNPV-infected larvae, is not a closed system, since larvae were feeding continuously during experiments. While infection was initiated immediately after the molt, it is expected that larval feeding increases between the day 1 and day 3 time points, concomitant with an acceleration of growth, which likely explains the changes in abundances of particular metabolites in control larvae over time (Figure 6, Figures S2–S4). However, it is well know that baculovirus-infected larvae also continue to feed and grow right up until they die, which is related to the expression of the baculoviral *egt* gene that regulates ecdysone metabolism [23]. Taking into account that larval feeding occurs at similar levels in infected and uninfected animals, it is believed that the comparison between both conditions at each time point is appropriate despite the observation that changes in abundance of metabolites can occur between day 1 and day 3 of larval feeding (which was not analyzed in this study).

Our analysis found that most DE metabolites are amino acids and that amino acid metabolism was the most enriched pathway in KEGG analysis (Figure 5, Figure S2). At the early stage of BmNPV infection, amino acids in the host cell are depleted, consistent with their consumption in large quantities and utilization in protein synthesis (Figure S2). On the other hand, an increase in abundance for most identified amino acids was detected at 3 dpi (Figure S2), for which it can be speculated that the baculovirus can reprogram host metabolism efficiently to meet the needs for enhanced protein synthesis during the peak of infection. The increase in amino acid metabolism caused by virus infection has been identified in other metabolomic studies of virus infection [6,24]. Amino acid metabolism increased during Newcastle disease virus infection in vivo and in vitro [6]. After cricket paralysis virus infection of silkworm-derived Bm5 cells, 17 of the 21 identified amino acids reached their highest level at 1 week post-infection and then gradually decreased [24]. Amino acids represent an important class of metabolites, that are not only utilized in the synthesis of proteins and other important biomolecules, but also provide intermediate metabolites for the TCA cycle and gluconeogenesis. The increased or decreased pools of amino acids are related to the demand for large-scale synthesis of viral proteins during viral replication.

However, the role of amino acid metabolism to support viral replication, with the exception of glutamine (a nonessential amino acid), is not well understood. Glutamine, fatty acids, and glucose are utilized in the cell as essential sources of carbon for synthesis of macromolecules and production of energy [25,26]. Current research on the interaction between virus replication and metabolic reactions has uncovered that glutaminolysis, glycolysis, and fatty acid metabolism play key roles in virus replication [1,3,27]. All these three processes are required at distinct stages of Kaposi’s sarcoma-associated herpesvirus and gallid alphaherpesvirus 1 infection [28,29]. Glutaminolysis and glycolysis are also essential for optimal replication of Marek’s Disease Virus [22]. However, vaccinia virus requires glutamine but not glucose for efficient replication [21].

In our study, on the other hand, no significant changes in metabolites related to glycolysis, TCA cycle, and fatty acid metabolism were observed in the hemolymph of silkworms after infection with BmNPV (Supplementary Data Sheet S4). Although it was found that amino acid metabolism plays an important role in BmNPV infection, the impact of glutamine metabolism was found to be minor. As a DNA virus with a large genome, the replication of BmNPV theoretically requires a large amount of basic materials and energy. While BmNPV infection does not show signs of reprogramming glycolysis, glutaminolysis, and fatty acid metabolism based on the global analysis of metabolites in the hemolymph, the importance of other metabolic pathways needs to be investigated in functional experiments. Our metabolome study can therefore become an important resource to guide further studies.

Interestingly, tryptophan metabolism has been believed to be closely related to the resistance of silkworms against BmNPV [30]. Qian et al. speculated that during BmNPV infection, tryptophan metabolism can be involved in arylhydrocarbon receptor (AhR) signaling and the activation of the silkworm immune system to inhibit viral infections [30]. Immune responses and metabolic regulation are tightly coupled in all animals including insects, but the underlying mechanistic connections are far from clear [31,32]. One example in mammals is the discovery of the interferon-induced enzyme cholesterol-25-hydroxylase, which produces oxysterols such as 25-hydroxycholesterol that regulate immune responses and exert broad antiviral effects [33]. Metabolism and immunity evolved in parallel but how the two systems interact with each other during virus infection requires more investigation. One approach would be the integration of different “omics” approaches, such as metabolomics and transcriptomics, which was pioneered in the present study. However, our integrated analysis of hemolymph metabolome and hemocyte transcriptome did not provide clear correlations between amino acid abundances and (immune) gene expression (Figure 8). It must be pointed out that only a minority of the metabolites in the hemolymph may have originated from hemocytes and that most metabolites that enter the circulatory system are produced by other tissues, mainly the fat body. This is considered to be an additional important feature of the experimental system that needs to be taken into account during the interpretation of the results, especially when making correlations between metabolome data in the hemolymph (affected by many tissues) and transcriptome data in hemocytes (that are cell-type-specific). In future studies, metabolome and other high-throughput (“omics”) technologies could be applied to other tissues, notably the fat body, which is a main target for baculovirus infection [34], and then be correlated with systemic changes in the metabolome of the hemolymph.

Interestingly, transcriptome and proteome approaches were also applied to investigate the impact of *Helicoverpa armigera* NPV infection of the fat body of *H. armigera* larvae [34]. Analysis indicated a large decrease in the expression of immune-related and metabolic (energy, carbohydrate, and amino acid) pathways at both the mRNA and protein level. In another recent study, extracellular adenosine signaling was implicated in the modulation of host metabolism and immune response during baculovirus infection of the silkworm [35]. During non-permissive infection of silkworm cells and larvae with *Autographa californica* NPV (AcMNPV), a much larger production of ATP, circulation of trehalose, and consumption of glucose was observed than after infection with BmNPV, which was interpreted as a mechanism for activation of the immune response rather than as a sign for the requirement of a large energy supply for baculovirus replication [35]. It was speculated that BmNPV can prevent adenosine receptor signaling and metabolic activation of the immune response through interference with the miRNA regulatory network in the host cells. Additionally, in the present study, extracellular adenosine was not detected as a DE metabolite in the hemolymph, consistent with the repression of this response by BmNPV infection.

Finally, our experiments using BmN cells found that the inhibitor of glycolytic 2-DG and the glutaminolysis inhibitor CB-839 did not inhibit BmNPV replication (Figure 9). These results confirm our metabolomics data, implying that BmNPV replication does not require glutaminolysis and glycolysis. Another recent study also found that glycolysis has no effect on the replication of BmNPV in permissive cells using 2-DG as inhibitor [35]. This phenomenon observed for baculovirus infections is different from that detected in infections of other viruses such as Dengue Virus [17], HSV-1 (herpes simplex virus 1), and influenza A [18]. The replication of these viruses requires glutaminolysis or glycolysis and their replication can be inhibited by 2-DG or CB-839. It should be noted that the Grace’s Insect Cell Culture Medium we used contains fructose. When 2-DG acts on BmN cells, some of the fructose may be able to compensate for entering the glycolytic pathway, which may interfere with our results to some degree.

## 5. Conclusions

Undoubtedly, viruses need to target host cell metabolism to ensure their efficient replication, and the host will also take several measures at various levels to counterbalance the use of its metabolites by the virus. We are at the initial stages of understanding the mechanisms of the interaction between virus infection and host metabolism and how they contribute to the course of infection. Our study provides an analysis of global metabolic profiling of BmNPV infection in hemolymph and highlights the importance of amino acid metabolism during BmNPV infection. Furthermore, the interaction between baculovirus and host may be differing from other viruses with respect to the requirements for glycolysis and glutaminolysis.

## Data Availability

RNA-sequencing data have been deposited in BioProject under the accession number PRJNA665856. For the metabolome data, since no suitable upload platform has been found, if anyone needs the original data, they can contact us at any time. We will provide him with the raw data.

## Supplementary Materials

The following are available online at https://www.mdpi.com/article/10.3390/v13050841/s1. Table S1: Identification and characterization of DE metabolites in silkworm hemocytes after BmNPV infection; Table S2: Detailed information on DE metabolite enrichment pathways; Table S3: Detailed information on DE metabolite-DEG pairs in the correlation analysis; Table S4: Information on metabolites and transporter genes involved in glycolysis, TCA cycle, glutamine metabolism and fatty acid metabolism; Figure S1: Principal component analysis (PCA) of hemolymph samples in the different experimental groups (control and BmNPV infection, 1 dpi and 3dpi); Figure S2: Box plots of amino acid changes in control and BmNPV-infected samples of silkworm hemolymph at 1 and 3 dpi; Figure S3: Box plots of carbohydrate changes in control and BmNPV-infected samples of silkworm hemolymph at 1 and 3 dpi; Figure S4: Box plots of nucleotide changes in control and BmNPV-infected samples of silkworm hemolymph at 1 and 3 dpi; Figure S5: DEGs in silkworm hemocytes infected with BmNPV at 1 and 3 dpi; Figure S6: Combined analysis of DE metabolite and DEG enrichment pathways; FigureS7: The network diagram of DE amino acid metabolites, metabolic pathways and the associated DEGs; FigureS8: The network diagram of DE carbohydrate metabolites, metabolic pathways and the associated DEGs; FigureS9: The network diagram of DE nucleotide metabolites, metabolic pathways and the associated DEGs; FigureS10: The network diagram of DE Lipid metabolites, metabolic pathways and the associated DEGs; Figure S11: Cytotoxicity assay of 2-deoxyglucose (2-DG) and CB-839.
