# Supplementary figures and images for: Global Metabolic Profiling of Baculovirus Infection in Silkworm Hemolymph Shows the Importance of Amino-Acid Metabolism
Source: Viruses. 2021 May 6;13(5):841. doi: 10.3390/v13050841 (PMC8148188; doi:10.3390/v13050841)

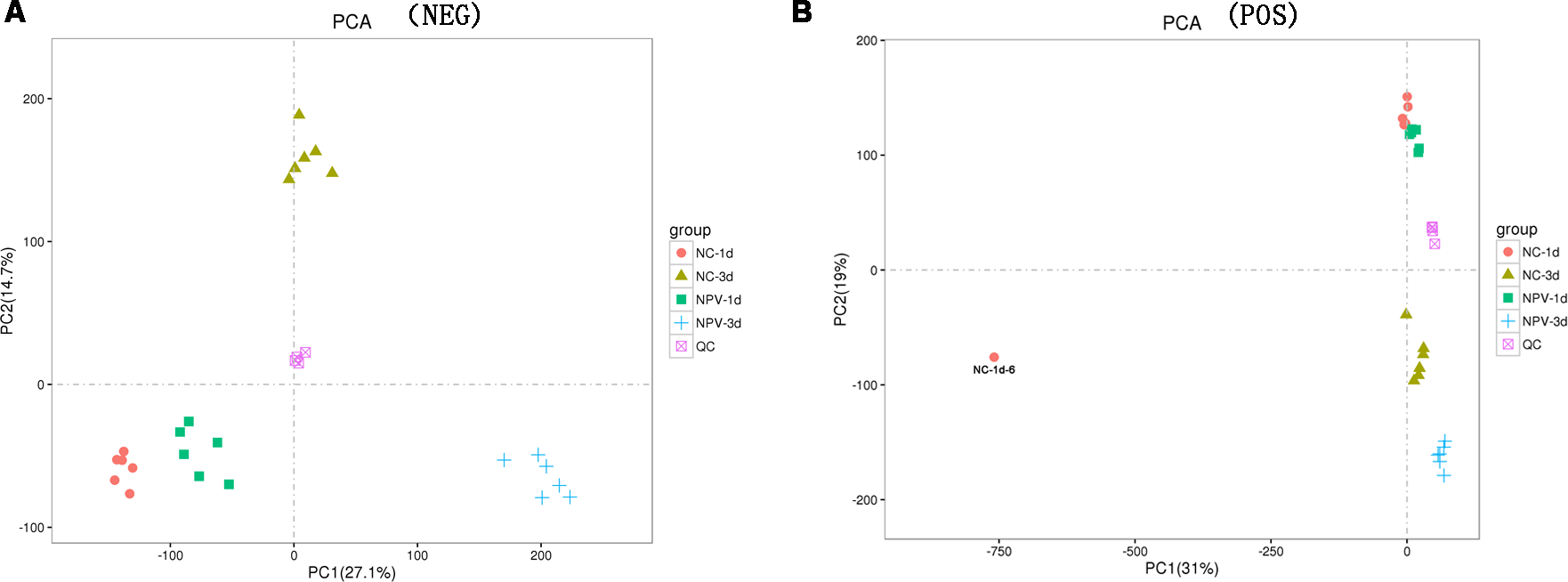

Supplement: Supplementary file 1 [file viruses-13-00841-s001.zip › Figure S1.tif]

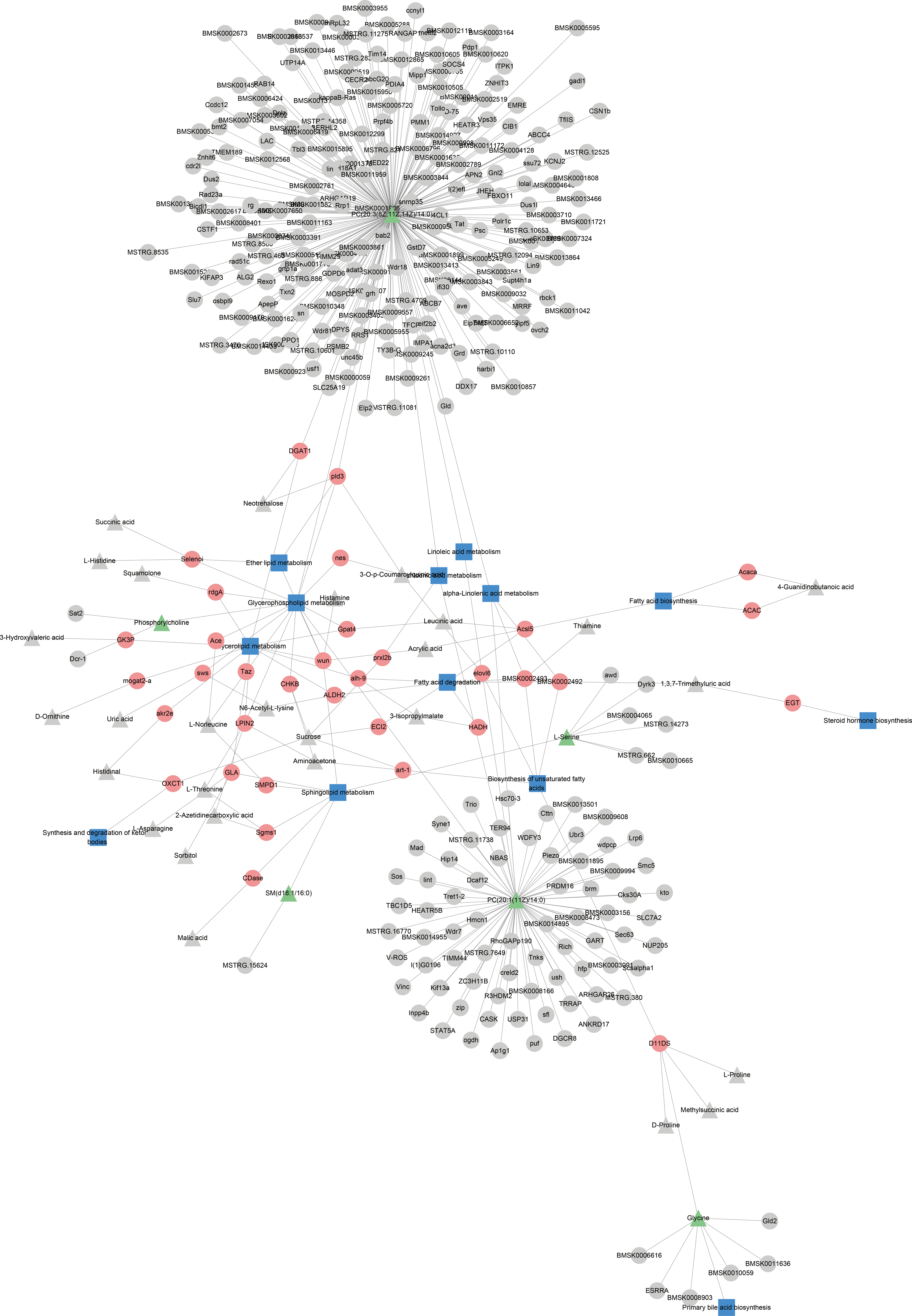

Supplement: Supplementary file 1 [file viruses-13-00841-s001.zip › Figure S10.tif]

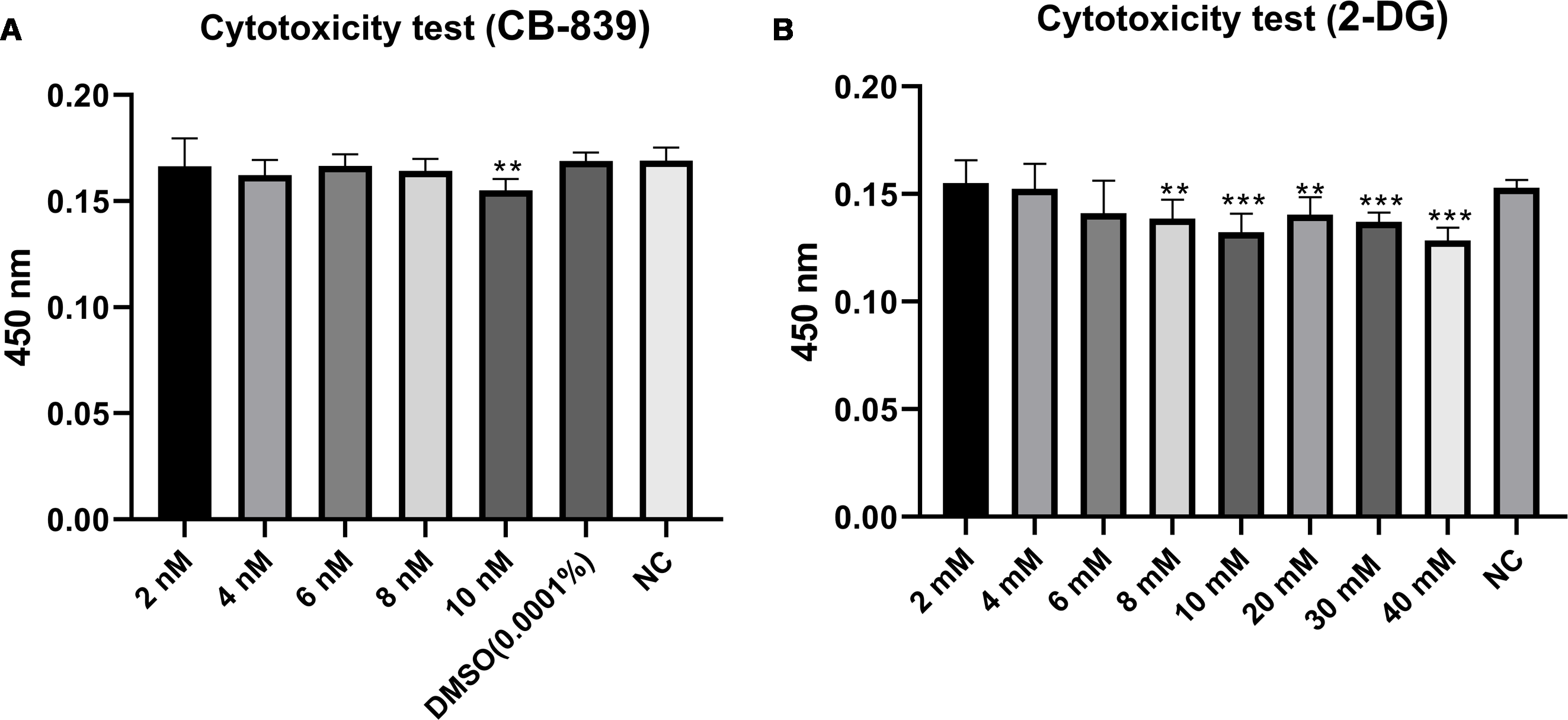

Supplement: Supplementary file 1 [file viruses-13-00841-s001.zip › Figure S11.tif]

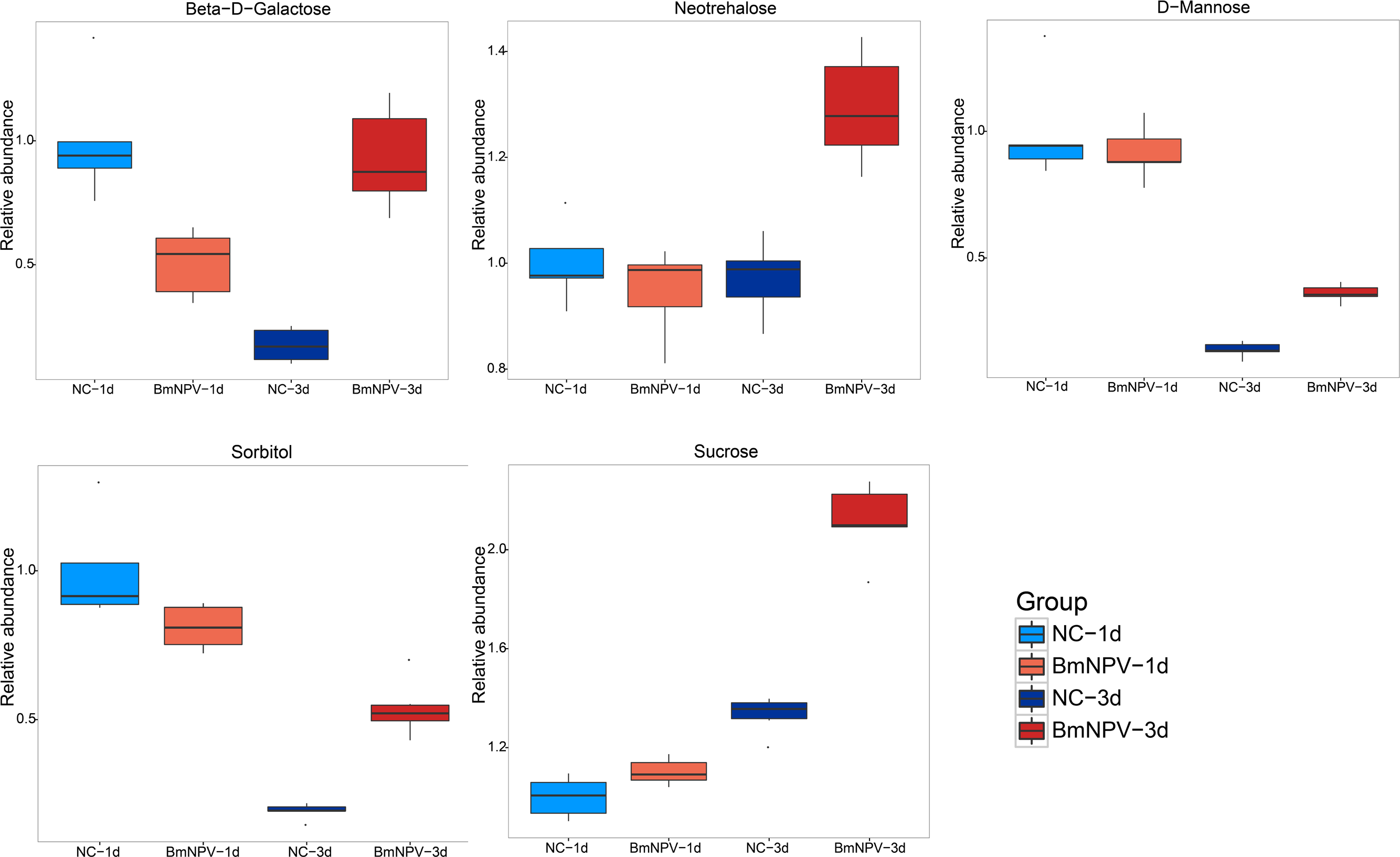

Supplement: Supplementary file 1 [file viruses-13-00841-s001.zip › Figure S3.tif]

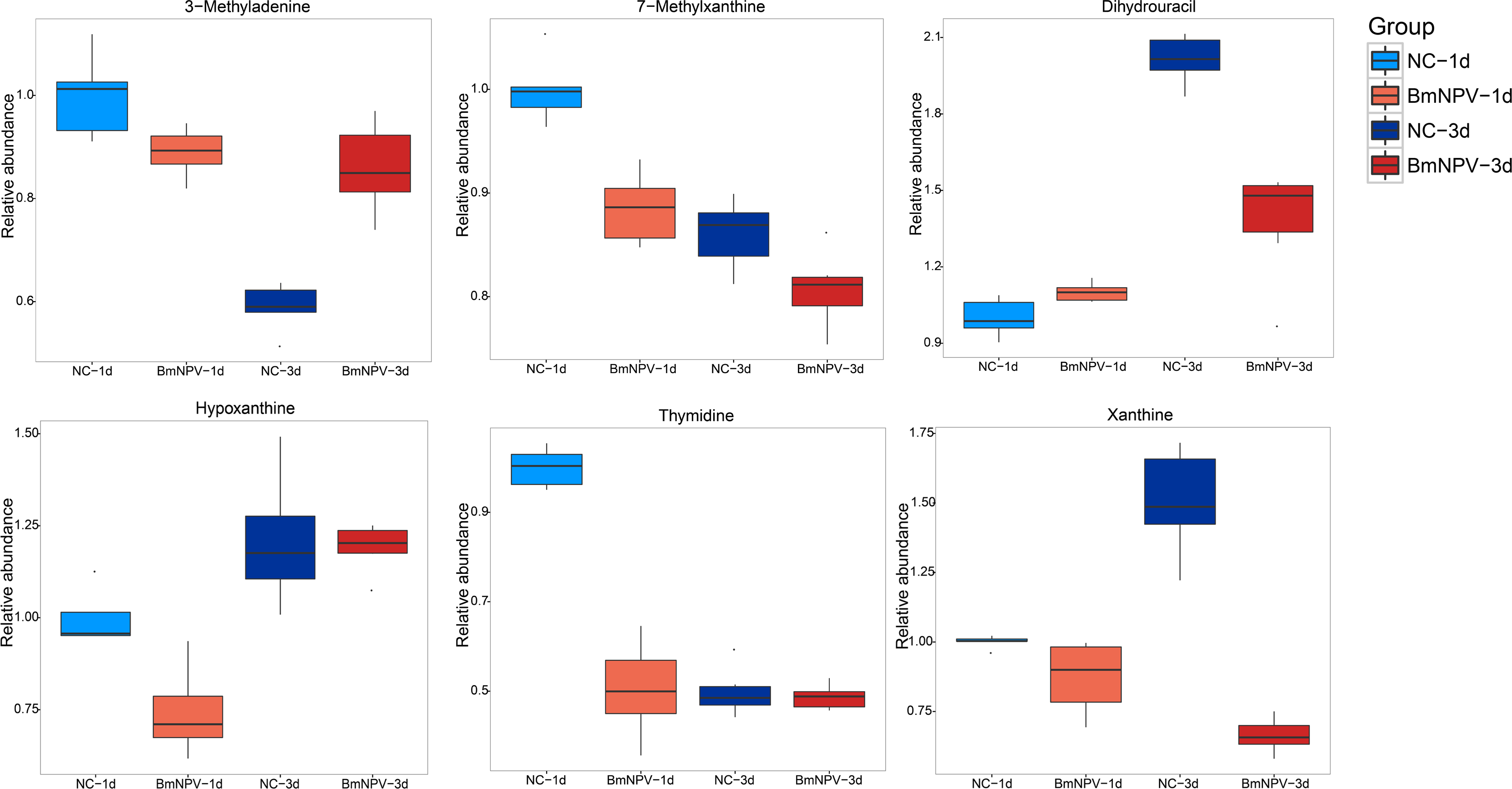

Supplement: Supplementary file 1 [file viruses-13-00841-s001.zip › Figure S4.tif]

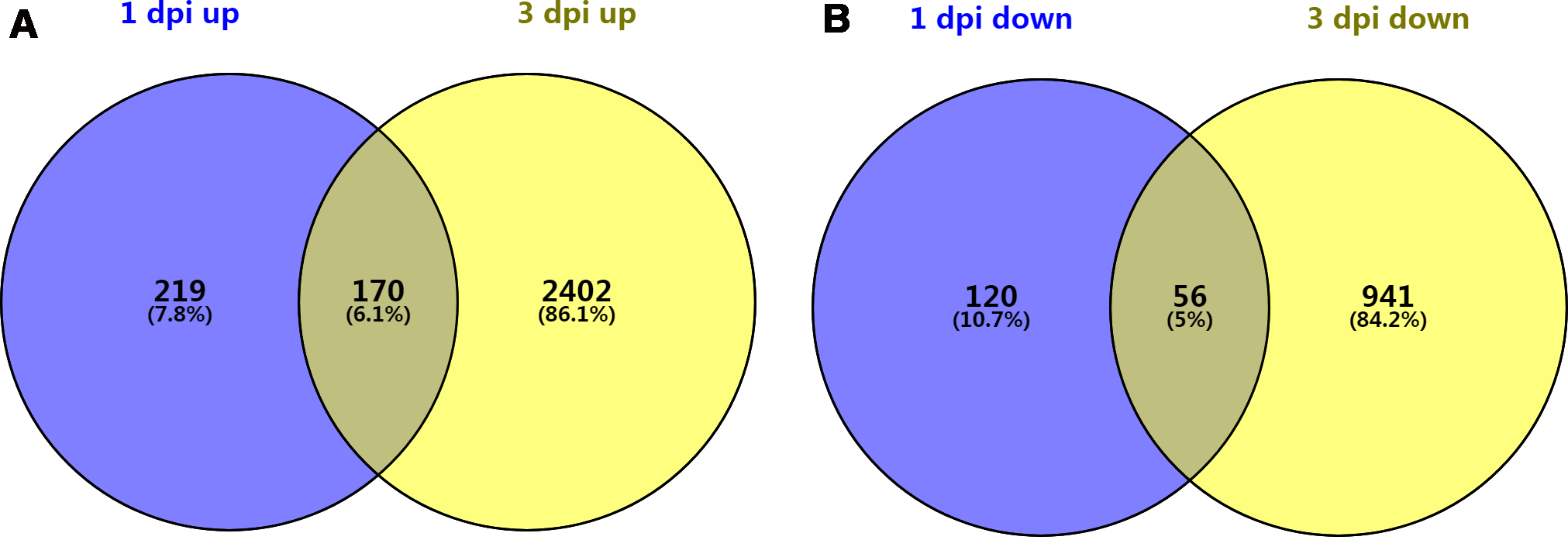

Supplement: Supplementary file 1 [file viruses-13-00841-s001.zip › Figure S5.tif]

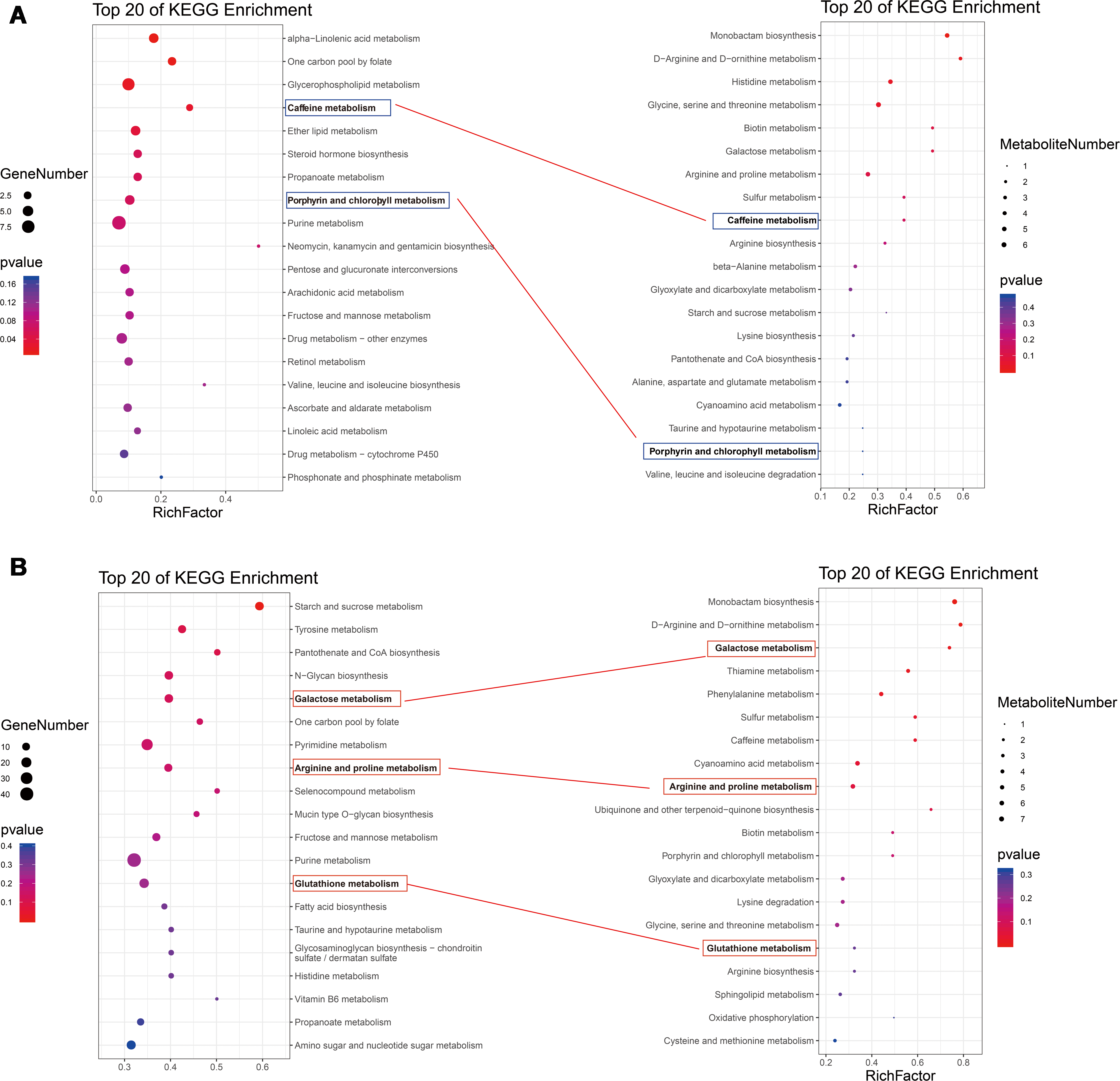

Supplement: Supplementary file 1 [file viruses-13-00841-s001.zip › Figure S6.tif]

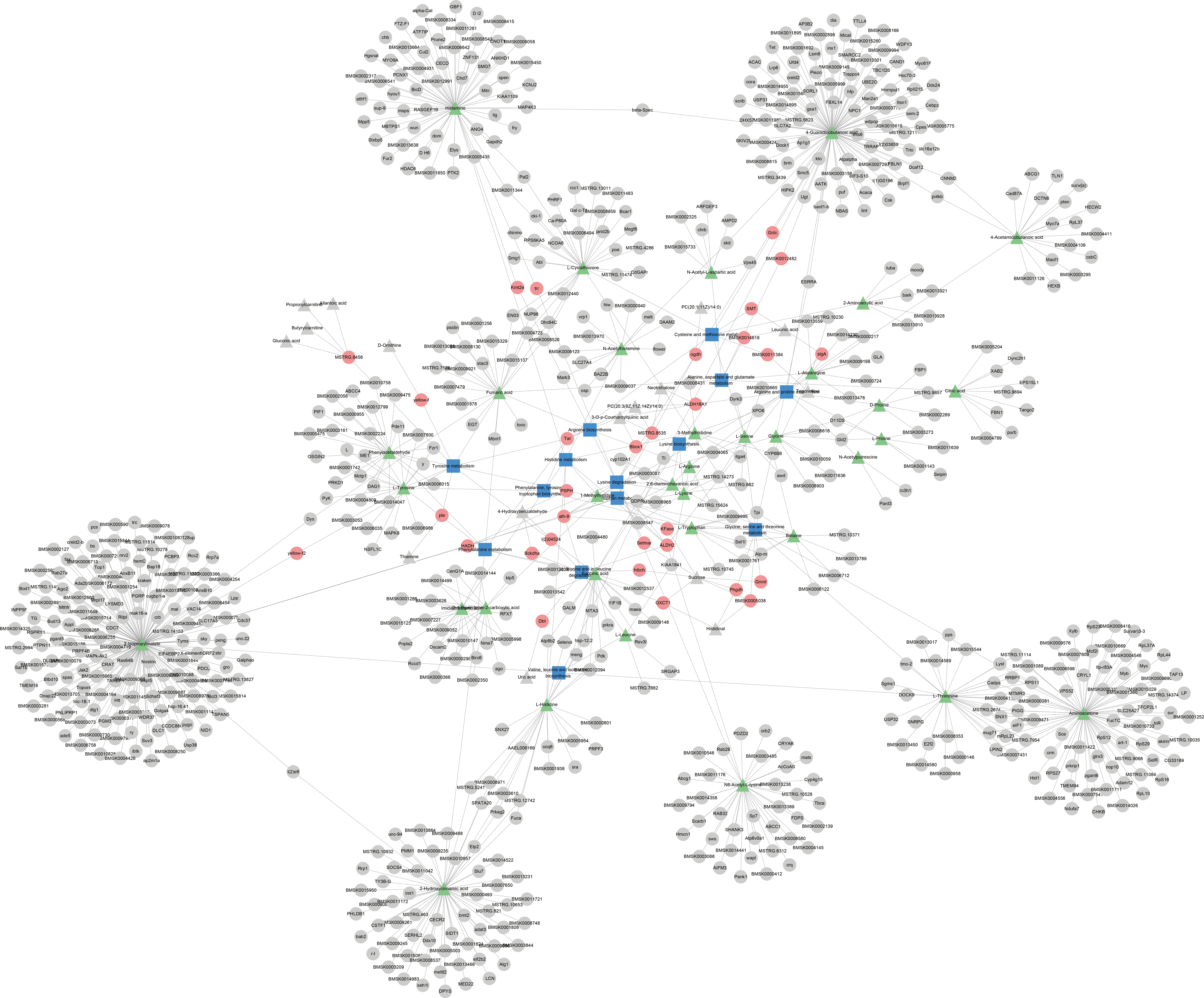

Supplement: Supplementary file 1 [file viruses-13-00841-s001.zip › Figure S7.tif]

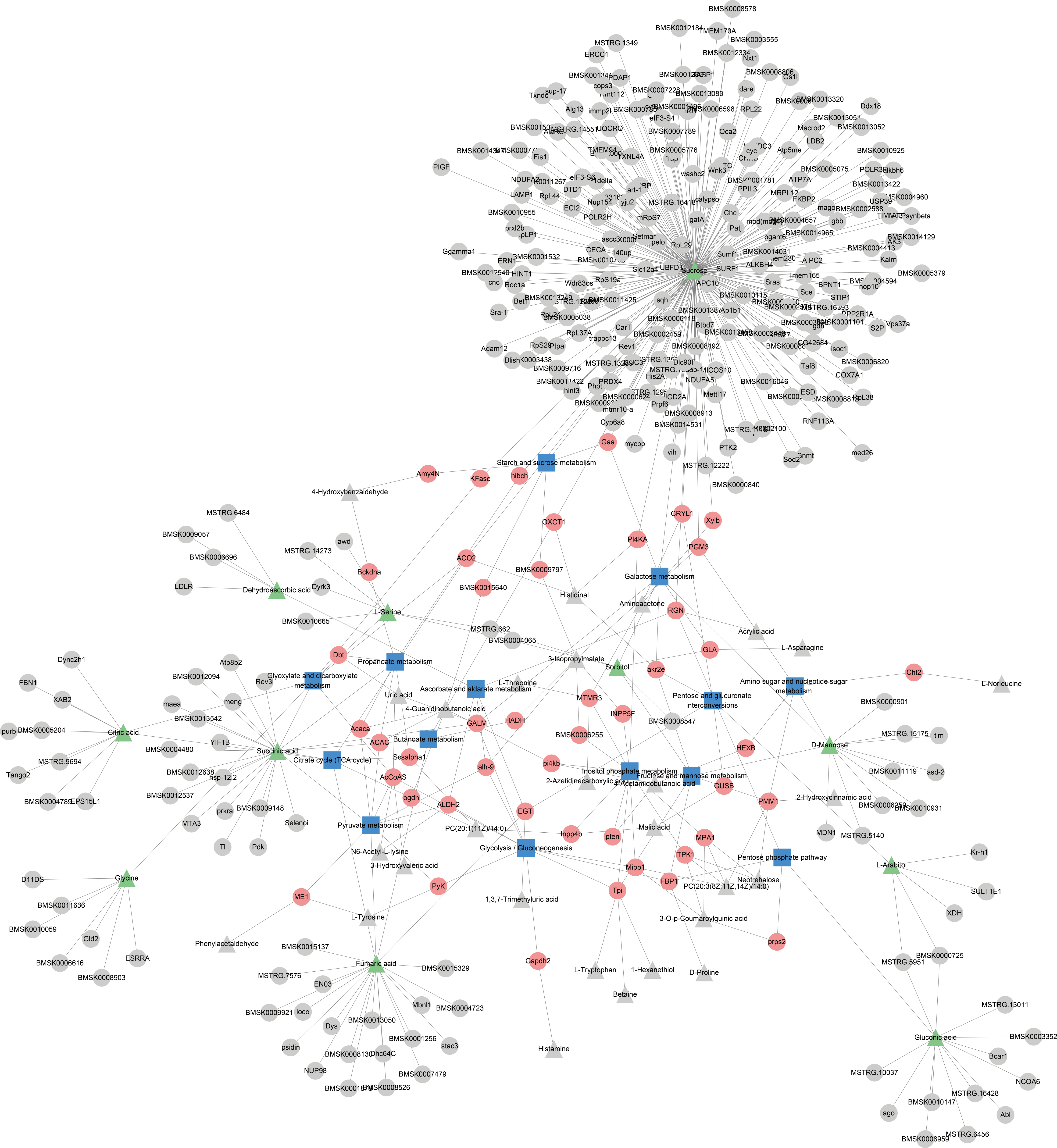

Supplement: Supplementary file 1 [file viruses-13-00841-s001.zip › Figure S8.tif]

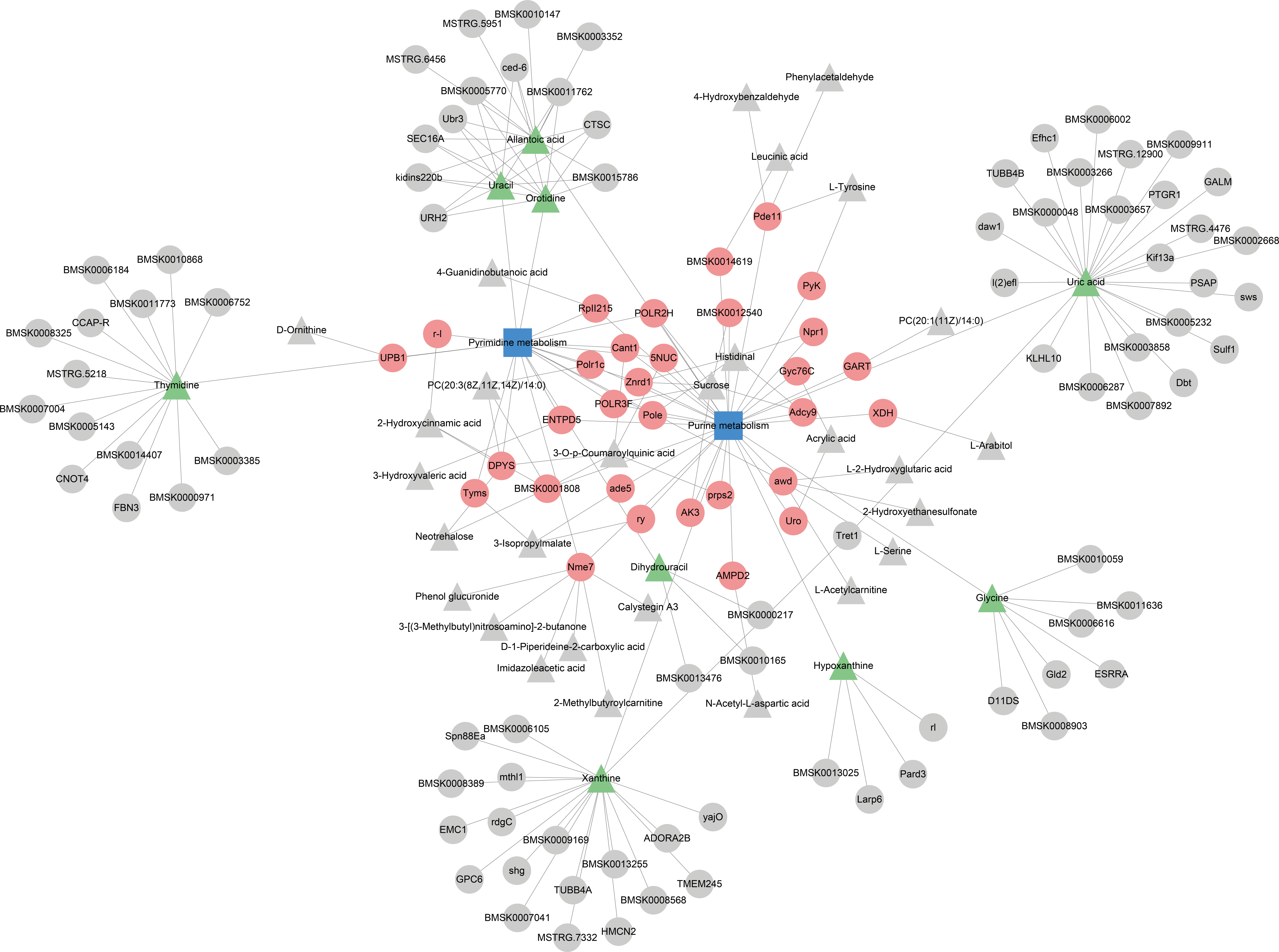

Supplement: Supplementary file 1 [file viruses-13-00841-s001.zip › Figure S9.tif]
